# HbA1c and height loss among Japanese workers: A retrospective study

**DOI:** 10.1371/journal.pone.0291465

**Published:** 2023-10-05

**Authors:** Yuji Shimizu, Hidenobu Hayakawa, Eiko Honda, Nagisa Sasaki, Midori Takada, Takeo Okada, Tetsuya Ohira, Masahiko Kiyama

**Affiliations:** 1 Division of Public Health, Osaka Institute of Public Health, Epidemiology Section, Osaka, Japan; 2 Department of Cardiovascular Disease Prevention, Osaka Center for Cancer and Cardiovascular Disease Prevention, Osaka, Japan; 3 Department of Epidemiology, Fukushima Medical University School of Medicine, Fukushima, Japan; Kindai University Faculty of Medicine, JAPAN

## Abstract

Evaluating the risk of height loss could be an efficient way to evaluate endothelial health, which might be associated with all-cause and cardiovascular mortality. Diabetes is an established risk factor both for intervertebral disk degeneration and osteoporosis-related fractures, which are major risk factors for height loss among adults. Therefore, hemoglobin A1c (HbA1c), as an indicator of the presence of diabetes, could be positively associated with height loss. A retrospective study of 10,333 workers aged 40 to 74 years was conducted. Height loss was defined as being in the highest quintile of height decrease per year. HbA1c in the normal range was positively associated with height loss. The known cardiovascular risk factors-adjusted odds ratio (OR) and 95% confidence interval (CI) for height loss with a 1-standard deviation (SD) increase in HbA1c (0.38% for both men and women) was 1.06 (1.02, 1.10) for men and 1.15 (1.07, 1.23) for women, respectively. When limit those analysis among those without diabetes, the magnitude was slightly higher; the fully adjusted OR and 95% CI for height loss with a 1-SD increase in HbA1c was 1.19 (1.11, 1.28) for men and 1.32 (1.20, 1.44) for women, respectively. Even when HbA1c is within the normal range, higher HbA1c is a significant risk factor for height loss among workers.

## Introduction

Since height loss is positively associated with all-cause and cardiovascular mortality [1–3], evaluating the risk of height loss could be an efficient way to evaluate endothelial health, which might be associated with all-cause and cardiovascular mortality.

Diabetes is an established risk factor both for intervertebral disk degeneration [4] and osteoporosis-related fractures [5], which are major risk factors for height loss among adults. Therefore, HbA1c, as an indicator of the presence of diabetes, could be positively associated with height loss.

In addition, a positive association between being in the normal range of HbA1c and cardiovascular risk factors has been reported among individuals without diabetes [6]. Being in the normal range of HbA1c could be positively associated with height loss among individuals without diabetes.

Furthermore, positive association between HbA1c and hypertension has been reported among individuals without diabetes [7]. Our previous study with Japanese workers revealed a significant positive association between hypertension and height loss, defined as the being in highest quintile of annual height decrease, for men but not for women [8].

However, no study reported the association between HbA1c and height loss among general population.

We hypothesized that HbA1c is significantly positively associated with height loss as defined as being in the highest quintile of height decrease per year among the general population. And we also hypothesized that even among participants without diabetes, this positive association could be observed.

To evaluate our hypothesis, we conducted a retrospective study of Japanese workers.

## Methods

### Study population

This study was conducted in accordance with the Declaration of Helsinki. It was approved by the ethics committee of Osaka Center for Cancer and Cardiovascular Diseases Prevention (Project registration code, R4-Rinri-4). To obtain consent for this study, the opt-out method was performed with a poster describing the study and subsequently an institutional website (www.osaka-ganjun.jp/effort/cvd/r-and-d/, accessed on 12 September 2023). Participants had the option to opt out of the study after viewing study information shared via poster (posted inside the facility) and institutional website. The authors had no access to information that could identify individual participants during or after data collection.

The Ministry of Health, Labour and Welfare of Japan started specific medical examinations for cardiovascular disease prevention in 2008. The present study population comprised 15,435 workers aged 40–74 years who participated in these medical examinations between 2008 and 2018 (baseline) at the Osaka Center for Cancer and Cardiovascular Diseases Prevention.

Participants without data on drinking status (n = 57), total cholesterol (TC) (n = 1,179), or HbA1c (n = 20) at baseline were excluded from the analysis. Participants without a height measurement during 2009–2019 (endpoint) were excluded from the analysis (n = 3,846). The remaining 10,333 subjects, with a mean age of 50.7 years (standard deviation [SD], 8.2 years; range, 40–74 years), were included in the study. The media follow up years was 3.1years (mean: 3.7 years and SD: 2.3 years). The total person-years was 38195.6 person years.

### Data collection and laboratory measurements

#### Baseline data

The baseline period of the present study was 2008–2018. Trained interviewers acquired information on history of cardiovascular disease, medication history, and habits. Briefly, height in feet while wearing stockings and weight while wearing light clothing were measured. BMI was calculated as weight divided by height squared (kg/m^2^). The coefficient of variation of height measurements were 3.45 for men (n = 6,623) and 3.48 for women (n = 3,710). To confirm the measurement value of height, a second measurement was taken when a difference of 3 cm or more was observed between current and previous measurements of height. Resting blood pressure was measured twice and the mean blood pressure was used in the analysis.

A fasting blood sample was collected. TC, triglycerides (TG), high-density lipoprotein cholesterol (HDLc), HbA1c, and serum creatinine were measured using standard procedures at the Osaka Center for Cancer and Cardiovascular Diseases Prevention. Low-density lipoprotein cholesterol (LDLc) was calculated using the Friedewald formula: LDLc = TC-(HDLc/5) mg/dL.

Between 2008 and 2012, HbA1c was measured using the Japanese Diabetes Society (JDS) definition. Starting in 2013, HbA1c was measured using the National Glycohemoglobin Standardization Program (NGSP) definition. The following equation, which was recently proposed by a JDS working group, was used to convert values: HbA1c (NGSP) = HbA1c (JDS) + 0.4% [9].

The World Health Organization (WHO) guidelines state that the international classification for high BMI among Asians is BMI ≥ 25 kg/m^2^ [10]. WHO also recommended BMI <18.5 kg/m^2^ to be considered low BMI among Asians [11]. We defined high BMI and low BMI according to the WHO recommendations.

Estimated glomerular filtration rate (eGFR) was calculated using an established method with a modification recently proposed by a working group of the Japanese Chronic Kidney Disease Initiative [12]. eGFR (mL/min/1.73 m^2^) was defined as 194 × (serum creatinine (enzyme method))^-1.094^× (age)^-0.287^ (×0.739 for women). Chronic kidney disease (CKD) was defined as eGFR < 60 mL/min/1.73 m^2^. Hypertension was defined as systolic blood pressure ≥140 mmHg, diastolic blood pressure ≥ 90 mmHg, or use of anti-hypertensive medication. Dyslipidemia was defined as TG ≥ 150 mg/dL, LDLc ≥ 140 mg/dL, HDLc < 40 mg/dL, or use of lipid-lowering medication. Diabetes was defined as HbA1c (NGSP) ≥ 6.5% or use of glucose-lowering medication.

#### Endpoint data

Height was measured during the endpoint period (2009–2019). A participant was considered to have height loss if he or she was in the highest quintile of height decrease per year, as in our previous studies (height loss/year ≥ 1.76 mm/year for men and height loss/year ≥ 2.04 mm/year for women) [8,13].

### Statistical analysis

Characteristics of the study participants were summarized by HbA1c level. Age was expressed as mean ± SD. Daily drinking, never drinker status, current smoker status, former smoker status, hypertension, high BMI, low BMI, dyslipidemia, and CKD were presented as percentages. Differences in those characteristics were calculated by HbA1c tertile.

Logistic regression was used to calculate odd ratios (ORs) and 95% confidence intervals (CIs) to determine associations between HbA1c and height loss.

Since we thought endothelial status might affect the association between HbA1c and height loss in the present study, known cardiovascular risk factors might act as confounders. However, since the present study evaluated the risk of height loss but not diseases related to height loss such as intervertebral disk degeneration and osteoporosis-related fractures, factors that are related to those diseases are mediators, not confounders. As a result, two models were generated. The first model adjusted only for sex and age (sex- and age-adjusted model). The second model (multivariable model) also included other established confounders that included established cardiovascular risk factors such as drinking status (non-drinker, often drinker, daily drinker), smoking status (never smoker, former smoker, current smoker), hypertension (yes, no), high BMI (yes, no), low BMI (yes, no), glucose-lowering medication (yes, no), dyslipidemia (yes, no), and CKD (yes, no).

Sex-specific associations between HbA1c and height loss was also calculated. To assess sensitivity, we also performed sex-specific analyses between HbA1c and height loss with height loss defined as being in the highest quartile of height decrease per year.

All statistical analyses were performed with SAS for Windows (version 9.4; SAS Inc., Cary, NC, USA); p values <0.05 were regarded as statistically significant.

## Results

### Characteristics of the study population by HbA1c tertile

Table 1 shows the characteristics of study population by HbA1c tertile. HbA1c tertile was significantly positively associated with age, never drinker status, hypertension, high BMI, dyslipidemia, and CKD and inversely associated with daily drinker status and low BMI.

**Table 1:** Characteristics of the study population.

	HbA1c tertile			P
	T1 (Low)	T2 (Middle)	T3 (High)	
No. at risk	3,233	3,752	3,348	
Men, %	66.5	61.2	65.0	<0.001
Age, years	48.5 ± 7.3	50.2 ± 8.2	53.3 ± 8.4	<0.001
Stroke, %	0.1	0.2	0.9	0.961
Ischemic heart disease, %	0.1	0.2	0.5	0.967
Daily drinker, %	24.4	18.8	17.0	<0.001
Never drinker, %	27.5	33.7	36.2	<0.001
Current smoker, %	26.2	26.2	27.5	0.362
Former smoker, %	31.1	30.0	30.6	0.636
Hypertension, %	21.7	26.1	36.9	<0.001
High BMI (≥25 kg/m^2^), %	19.1	24.6	37.6	<0.001
Low BMI (<18.5 kg/m^2^), %	8.1	6.8	5.1	<0.001
Dyslipidemia, %	35.5	45.5	56.4	<0.001
CKD, %	6.7	10.1	14.7	<0.001
Glucose-lowering medication use, %	0.0	0.3	11.2	<0.001

Age: Mean ± standard deviation. Height loss was defined as being in the highest quintile of height decrease per year. For men, HbA1c tertiles were <5.3% for T1 (Low), 5.3–5.5% for T2 (Middle), and 5.6%≤ for T3 (High). For women, the corresponding values were <5.2%, 5.2–5.5%, and 5.6≤. For both men and women, 1 standard deviation for HbA1c was 0.38%. HbA1c: Hemoglobin A1c, BMI: Body mass index, CKD: Chronic kidney disease.

### Associations between HbA1c and height loss

As shown in Table 2, HbA1c was positively associated with height loss. The age-adjusted OR and 95% CI of height loss for a 1-SD increase in HbA1c was 1.19 (1.11, 1.28). After adjusting for known cardiovascular risk factors, the OR was slightly lower but remained significant. The OR and 95% CI from the multivariable model was 1.06 (1.02, 1.10).

**Table 2:** Associations between HbA1c and height loss.

	HbA1c tertile			p	1 SD increase in HbA1c
	T1 (Low)	T2 (Middle)	T3 (High)		
Total					
No. of participants	3,233	3,752	3,348		
Person-years	14135.9	13364.6	10695.1		
No. of height loss (%)	457 (14.1)	792 (21.1)	817 (24.4)		
Sex- and age-adjusted OR	Referent	1.54 (1.35, 1.74)	1.68 (1.47, 1.91)	<0.001	1.19 (1.11, 1.28)
Multivariable OR	Referent	1.55 (1.37, 1.77)	1.69 (1.48, 1.94)	<0.001	1.06 (1.02, 1.10)

Multivariable odds ratios (ORs) were adjusted for sex and age, drinking status, smoking status, hypertension, glucose-lowering medication, dyslipidemia, chronic kidney disease, and high body mass index (BMI). Height loss was defined as being in the highest quintile of height decrease per year. For men, HbA1c tertiles were <5.3% for T1 (Low), 5.3–5.5% for T2 (Middle), and 5.6%≤ for T3 (High). For women, the corresponding values were <5.2%, 5.2–5.5%, and 5.6≤. For both men and women, 1 standard deviation (SD) for HbA1c was 0.38%.

### Sex-specific associations between HbA1c and height loss

Table 3 shows sex-specific associations between HbA1c and height loss. The associations for men and women were essentially the same. For both men and women, a significant positive association between HbA1c and height loss were observed.

**Table 3:** Sex-specific associations between height loss and HbA1c.

	HbA1c tertile					p	1 SD increase in HbA1c
	T1 (Low)		T2 (Middle)	T3 (High)			
Men							
No. of participants	2,150	2,297			2,176		
Person-years	9504.3	8330.7			7389.3		
No. of height loss (%)	320 (14.9)	504 (21.9)			500 (23.0)		
Age-adjusted OR	Referent	1.63 (1.38, 1.92)			1.64 (1.39, 1.93)	<0.001	1.14 (1.05, 1.23)
Multivariable OR	Referent	1.64 (1.39, 1.93)			1.63 (1.37, 1.94)	<0.001	1.06 (1.02, 1.10)
Women							
No. of participants	1,083	1,455			1,172		
Person-years	4631.6	5033.9			3305.8		
No. of height loss (%)	137 (12.7)	288 (19.8)			317 (27.0)		
Age-adjusted OR	Referent	1.55 (1.24, 1.93)			2.06 (1.64, 2.59)	<0.001	1.41 (1.21, 1.64)
Multivariable OR	Referent	1.55 (1.24, 1.95)			2.02 (1.60, 2.56)	<0.001	1.15 (1.07, 1.23)

Multivariable odds ratios (ORs) adjusted further for age, drinking status, smoking status, hypertension, glucose-lowering medication. dyslipidemia, chronic kidney disease, and high body mass index (BMI). Height loss was defined as being in the highest quintile of height decrease per year. For men, HbA1c tertiles were <5.3% for T1 (Low), 5.3–5.5% for T2 (Middle), and 5.6%≤ for T3 (High). For women, the corresponding values were <5.2%, 5.2–5.5%, and 5.6≤. For both men and women, 1 standard deviation (SD) for HbA1c was 0.38%.

The age-adjusted OR and 95% CI for height loss with a 1-SD increase in HbA1c were 1.14 (1.05, 1.23) for men and 1.41 (1.21, 1.64) for women, respectively. After adjusting for known cardiovascular risk factors, ORs were slightly lower but remained significant. The ORs and 95% CIs from the multivariable model were 1.06 (1.02, 1.10) for men and 1.15 (1.07, 1.23) for women, respectively.

### Sex-specific associations between HbA1c and height loss among participants without diabetes

Table 4 shows sex-specific associations between HbA1c and height loss among participants without diabetes. The associations for men and women were essentially the same. For both men and women, a significant positive association between HbA1c and height loss were observed. The known cardiovascular risk factors-adjusted OR and 95% CI for height loss with a 1-SD increase in HbA1c were 1.19 (1.11, 1.28) for men and 1.32 (1.20, 1.44) for women, respectively.

**Table 4:** Sex-specific associations between height loss and HbA1c among participants without diabetes.

	HbA1c tertile					p	1 SD increase in HbA1c
	T1 (Low)	T2 (Middle)			T3 (High)		
Men							
No. of participants	2,150		2,289	1,604			
Person-years	9504.3		8302.1	5328.9			
No. of height loss (%)	320 (14.9)		504 (22.0)	355 (22.1)			
Age-adjusted OR	Referent		1.55 (1.33, 1.81)	1.46 (1.23, 1.74)		<0.001	1.53 (1.28, 1.82)
Multivariable OR	Referent		1.59 (1.36, 1.86)	1.50 (1.26, 1.79)		<0.001	1.19 (1.11, 1.28)
Women							
No. at participants	1,082		1,453	1,081			
Person-years	5574.3		3323.5	2983.2			
No. of height loss (%)	136 (12.6)		287 (19.8)	295 (27.3)			
Age-adjusted OR	Referent		1.55 (1.24, 1.94)	2.11 (1.68, 2.66)		<0.001	2.09 (1.66, 2.63)
Multivariable OR	Referent		1.56 (1.24, 1.96)	2.09 (1.65, 2.65)		<0.001	1.32 (1.20, 1.44)

Multivariable odds ratios (ORs) adjusted further for age, drinking status, smoking status, hypertension, dyslipidemia, chronic kidney disease, and high body mass index (BMI).

Height loss was defined as being in the highest quintile of height decrease per year.

For men, HbA1c tertiles were <5.3% for T1 (Low), 5.3–5.5% for T2 (Middle), and 5.6%≤ for T3 (High). For women, the corresponding values were <5.2%, 5.2–5.5%, and 5.6≤. For both men and women, 1 standard deviation (SD) for HbA1c was 0.38%.

### Sensitivity analysis

To assess sensitivity, we performed the analyses for HbA1c and height loss again with height loss defined as being in the highest quartile of height decrease per year. We obtained essentially the same results. In the multivariable model, with T1 (Low) as the referent group, the ORs for height loss among men were 1.40 (1.22, 1.62) for T2 and 1.35 (1.16, 1.58) for T3. Among women, the corresponding values were 1.42 (1.24, 1.64) and 1.75 (1.41, 2.17), respectively. And among those without diabetes, with T1 (Low) as the referent group, the ORs for men were 1.42 (1.24, 1.64) for T2 and 1.32 (1.13, 1.55) for T3. For women, the corresponding values were 1.44 (1.17, 1.76) and 1.79 (1.44, 2.22), respectively.

## Discussion

The major finding of the present study is that HbA1c is positively associated with height loss among Japanese workers. Furthermore, even when HbA1c is within the normal range, higher HbA1c is a significant risk factor for height loss among workers.

The mechanism underlying the positive association between HbA1c and height loss found in the present study is currently unknown. Since height loss is positively associated with all-cause and cardiovascular mortality [1–3], endothelial status might be underlying this association.

Our previous retrospective study of 11,154 Japanese individuals aged 40–74 years investigated the association between hypertension and height loss defined as being in the highest quintile of height decrease per year. This previous study showed that independent of known confounding factors, baseline hypertension is a significant risk factor for height loss only among men [8]. Since hypertension is positively associated with endothelial dysfunction [14], unfavorable endothelial status might induce height loss.

We performed another retrospective study of 9,651 Japanese workers aged 40–74 years that revealed a significant inverse association between hemoglobin levels and height loss in men with BMI <25 kg/m^2^, but not in men with BMI ≥25 kg/m^2^ [13]. In this previous study, BMI ≥ 25kg/m^2^ was positively associated with height loss [13]. Hemoglobin is also reported to be positively associated with hypertension and positively associated with atherosclerosis as evaluated by cardio-ankle vascular index (CAVI) for participants with BMI <25 kg/m^2^ but not for those with BMI ≥ 25kg/m^2^ [15,16]. Therefore, even though higher hemoglobin levels are associated with hypertension-induced vascular damage [17], hemoglobin reduces oxidative stress and hypoxia, which might have the beneficial effect of preventing height loss, possibly by maintaining endothelial function.

Reticulocytes, which are immature red blood cells, are a source of hemoglobin that is positively associated with hypertension but inversely associated with endothelial dysfunction [18]. This study found that oxidative stress might induce hypertension and stimulate reticulocyte production but reticulocyte reduces oxidative stress, which reduces the risk of developing endothelial dysfunction.

Those studies indicate that the beneficial influence of maintaining the endothelium might prevent height loss. However, how the status of the endothelium influences vertebral fractures and decreases in intervertebral disc height, which are major causes of height loss among adults, remains unknown.

Hypoxia accelerates intervertebral disc degeneration [19,20]. A previous study reported a significant inverse association between height loss and CD34-positive cell count [21]. Since CD34-positive cells contribute to maintenance of the endothelium [22,23] and angiogenesis [24], inadequate angiogenesis related to lower adaptability to hypoxia might increase the risk of height loss via the development of intervertebral disc degeneration. Due to the inverse association reported between circulating CD34-positive cell count and HbA1c [25], HbA1c might act as an indicator of adaptability to hypoxia, even when HbA1c is within the normal range.

Diabetes, which is related to high HbA1c, is an established risk factor for both intervertebral disk degeneration [4] osteoporosis-related fractures [5]. Furthermore, a previous study with men aged 65 years or over reported a significant positive association between HbA1c levels within the normal range and osteoporotic fractures [26]. Since osteoporotic fracture is a known cause of height loss among adults, those studies partly support our present results; being in the normal range of HbA1c is positively associated with height loss among participants without diabetes.

In the present study, we found that HbA1c is significantly positively associated with height loss among Japanese workers without diabetes, even when HbA1c is within the normal range. After adjusting for known cardiovascular risk factors, the magnitude of the association was slightly lower but the association remained significant. Therefore, even when within the normal range, HbA1c could be associated with height loss.

In older [3] and middle-aged [1] individuals, height loss was reported to be associated with high mortality in later life. Therefore, the present findings could help clarify the mechanism underlying the relationship between height loss and all-cause mortality. This is a strength of the present study. A clinical implication of the present study is that HbA1c in the normal range could be an efficient way to estimate the risk of height loss, which might be related to endothelial status.

Fast eating speed is associated with height loss among Japanese workers [27], but eating speed might not determine the association between HbA1c and height loss. In the present study population, data on eating speed was available for 7,950 participants. There were no significant associations between eating speed and HbA1c. The mean ±SD HbA1c was 5.5 ± 0.6% for slow eaters (n = 637), 5.5 ± 0.6% for moderate speed eaters (n = 4,213), and 5.4 ± 0.6% for fast eaters (n = 3,100) (p = 0.136).

Because we do not know the efficient cutoff point for evaluating height loss, the correlation between annual height decreases as a continuous variable and HbA1c was also calculated. The partial correlation coefficient (r) between annual height decreases and HbA1c was r = 0.02 (p = 0.020) in the sex- and age-adjusted model and r = 0.02 (p = 0.019) in the multivariable model. Although there were statistically significant differences, the difference is not biologically relevant because it is smaller than variations in height throughout the day [28]. To reduce the influence of diurnal variations in height, we used a binary model to define height loss, like in our previous studies [8,13,21].

The potential limitations of this study warrant consideration. Intervertebral disc degeneration and vertebral fractures associated with osteoporosis might play an important role in height loss among adults, but those data were not available to us, as in our previous studies [8,13,21]. Many individuals with intervertebral disc degeneration do not experience pain [29]. Most patients with vertebral fractures are asymptomatic and diagnosed incidentally [30]. To identify those diseases among the general population, plain radiographs, computed tomography, or magnetic resonance imaging is necessary. An efficient cutoff point to define height loss has not been established. In the present study, we defined height loss as being in the highest quintile of height decrease per year. However, our sensitivity analysis based on quartile of height decrease per year showed essentially the same associations. Circulating CD34-positive cell count might affect the association between HbA1c and height loss found the in present study, but we have no data about circulating CD34-positive cell counts. Circulating CD34-positive cell count could be influenced by both genetic factors and exercise [31,32]. Further investigation with circulating CD34-positive count and data related to genetic factors and exercise is necessary to clarify the influence of CD34-positive cells on the present association.

## Conclusion

In conclusion, independent of known cardiovascular risk factors, higher HbA1c, even when within the normal range, is a significant risk factor for height loss among Japanese workers.
